# Cerebrospinal fluid proteomics reveal potential protein targets of JiaWeiSiNiSan in preventing chronic psychological stress damage

**DOI:** 10.1080/13880209.2021.1954666

**Published:** 2021-08-12

**Authors:** Han-Zhang Wang, Wu-Long Luo, Ning-Xi Zeng, Hui-Zhen Li, Ling Li, Can Yan, Li-Li Wu

**Affiliations:** Research Center for Basic Integrative Medicine, Guangzhou University of Chinese Medicine, Guangzhou, China

**Keywords:** Stress resilience, hippocampus neurogenesis, neuron loss, Chronic Unpredictable Mild Stress model

## Abstract

**Context:**

Chinese herbal formula JiaWeiSiNiSan (JWSNS) has been widely used to prevent stress-induced neuropsychiatric ailments in clinics and proven to have therapeutic anti-stress effects on rats. However, the mechanism remains unclear.

**Objective:**

Based on the proteomics of cerebrospinal fluid (CSF), this study explores the possible mechanism and target proteins of JiaWeiSiNiSan raising stress resilience and preventing stress damage.

**Materials and methods:**

A 6-week Chronic Unpredictable Mild Stress (CUMS) model was applied on adult Wistar male rats to observe the effects of JWSNS on improving mental stress resilience. Tandem Mass Tag (TMT) proteomics and bioinformatics analysis were used to screen and analyze differentially expressed proteins (DEPs) in CSF. Parallel Reaction Monitoring (PRM) was used to validate target DEPs.

**Results:**

Significantly decreased sucrose preference, locomotion activity level and accuracy of T-maze, as well as increased immobility time, were observed in CUMS rats compared to CON rats while JWSNS improved above depression-like behaviours. The quantitative proteomics and bioinformatics analysis showed that JWSNS decreased the expression of Rps4x, HSP90AA1, Rps12, Uba1, Rsp14, Tuba1b in CUMS rats CSF (*p* < 0.05, FDR < 0.5). Immunofluorescence results showed that the number of BrdU/DCX positive cells (*p* < 0.01) and the relative number of neurons (*p* < 0.01) in the hippocampus dentate gyrus (DG) of the JSWNS group significantly increased, compared with the CUMS group.

**Conclusions:**

JWSNS could increase mental stress resilience and prevent stress damage by regulating proteins in CSF. This study provides a scientific basis for further study on Chinese formulas preventing mental illness.

## Introduction

Mental stress injury could trigger the onset of neuropsychiatric ailments, such as major depression (MDD), post-traumatic stress disorder (PTSD), late-onset depression (LOD), and anxiety, with clinical manifestations of decreased learning and memory ability, depressive behaviours and suicidal tendency, which seriously affect normal life and work. People respond to stressors in very different ways. Part of people may feel threatened by stressful challenges develop mental illness, while some individuals are able to cope with even severe stressors and keep stress-resilient – they are relatively unaffected by external factors (stressors) and could recover from mental stress damage physiological mental stress. Stress resilience refers to the ability of individuals to quickly recover from and adapt to stress and difficulties (Luthar et al. 2000). Whether one is resilient or susceptible to stress is not only congenital but also adjustable by environment and training. Improving stress resilience (of people susceptible to stress) is of importance for preventing psychological stress-related diseases.

Current studies on neuropsychiatric diseases mainly focus on how to control and relieve the symptoms with drugs. To prevent morbidity, patients often get help from non-pharmacological methods, such as mental health education, psychological counselling and mindfulness meditation. Traditional Chinese Medicine attaches importance to disease prevention and has a history of psychological state adjustment for more than 2000 years.

**Figure 1.**
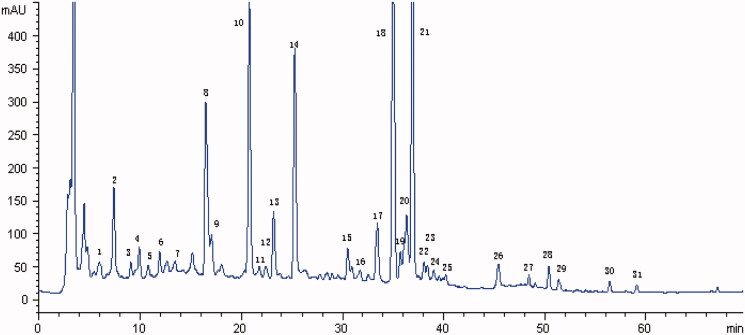
HPLC fingerprint graph of JWSNS. The HPLC fingerprint graph of JWSNS showed 31 common characteristic peaks: one peak (1) belonged to *Rehmannia glutinosa*; one peak (7) belonged to *Lycium barbarum* L.; eight peaks (3, 4, 5, 6, 9, 10, 20, 23 and 25) belonged to *Gardenia jasminoides* Ellis; eight peaks (2, 8, 12, 13, 14, 16, 20 and 27) belonged to *Paeonia lactiflora*; 14 peaks (11, 15, 17, 18, 19, 20, 21, 22, 24, 26, 28, 29, 30 and 31) belonged to *Aurantii Fructus*. Compared with reference substance, peaks 10, 14, 18 and 19 were respectively identified as geniposide, paeoniflorin, naringin, hesperidin.

Similar to health care products, Chinese patent drugs often require long-term administration of small doses. Chinese herbal compound JiaWeiSiNiSan (JWSNS), also known as Modified SiNiSan, evolved from a classic Chinese Medicine formula – SiNiSan, a widely used mental adjustment formula (Shen et al. 2020) which was first recorded in ‘Shang Han Lun’ (25–220 AD) – a classic of TCM. Based on the basic theory and pharmacology of Chinese medicine, JWSNS has been commonly applied in treating mental disorders such as depression and anxiety for thousands of years (Cai et al. 2021). JWSNS is composed of *Bupleurum chinense* DC**. (**Apiaceae) root (Chaihu), *Paeonia lactiflora* Pall. (Paeoniaceae) root (Baishao), *Citrus × aurantium* L. (Rutaceae) unripe fruit (Zhiqiao), *Lycium barbarum* L. (Solanaceae) ripe fruit (Gouqi), *Gardenia jasminoides* J.Ellis (Rubiaceae) ripe fruit (Zhizi), *Rehmannia glutinosa* Seud (Scrophulariacea) rootstock (Dihuang) and *Haliotis diversicolor* Reeve (Haliotidae) (Shijueming) (as shown in Table 1). A number of studies have proven the therapeutic effects of JWSNS on ameliorating stress-induced depressive behaviours by acting on HPA-axis (Shi et al. 2008), hippocampus (Wang et al. 2007), amygdala (Yan et al. 2005), as well as plasma and adrenal gland (Xie et al. 2013). Modern pharmacological studies have shown that the main active ingredients of JWSNS are paeoniflorin, pericarpiin, geniposide, naringin, and hesperidin (Maratha and Mahadevan 2012; Zhong et al. 2018; Ben-Azu et al. 2019; Chen et al. 2019; Fu et al. 2019; Liu et al. 2011; Liu et al. 2019; Kwatra et al. 2020) (Figure 1). But the mechanism in JWSNS preventing and treating stress injury diseases remains unclear. Due to the multi-component and multi-target characteristics of Chinese medicine, it is important to choose appropriate objects and regions to explore the mechanism of JWSNS regulating stress response.

**Table 1.** The table of components in JiaWeiSiNiSan.

Plant species	Family	Plant part	Pinyin
*Bupleurum chinense* DC.	Apiaceae	Root	Chai hu
*Paeonia lactiflora* Pall.	Paeoniaceae	Root	Bai shao
*Citrus × aurantium* L.	Rutaceae	Fruit	Zhi qiao
*Lycium barbarum* L.	Solanaceae	Fruit	Gou qi
*Gardenia jasminoides* J.Ellis	Rubiaceae	Fruit	Zhi zi
*Rehmannia glutinosa* Seud.	Scrophulariacea	Rootstock	Di huang
*Haliotis diversicolor Reeve*	Haliotidae	Shell	Shi jue ming

Psychological stress response could not only regulate and injury multiple brain regions (such as the hippocampus, amygdala, hypothalamus, and prefrontal cortex) but also affect the peripheral system. Recently, the boundary areas of the brain, such as meninges, cerebrospinal fluid, choroid plexus that connect the brain and periphery have become a hotspot of research on stress. CSF could provide a source of neurological and psychiatric biomarkers (Al Shweiki et al. 2017) – including products of nerve cell shedding and CNS-derived proteins. In a large number of studies on stress-related diseases (including depression and PTSD), researchers have found that the contents of different proteins in the CSF of patients have been changed (Baker et al. 1999; Sah et al. 2009; Bonne et al. 2011; Martinez et al. 2012). At the same time, these changes were reversed after drug treatment (Mathé et al. 2007; Madaan and Wilson 2009), indicating that CSF could reflect the antistress damage effect of drugs. To sum up, CSF may be a good detection and research object in terms of both the systemic characteristics of stress response and the universality of the active site of JWSNS.

Therefore, we hypothesized that JWSNS could prevent chronic psychological stress damage through CSF. In this study, we applied a 6-week CUMS model to simulate chronic psychological stress injury and multiple behavioural tests such as open field test (OFT), sucrose preference test (SPT), forced swimming test (FST) and T maze to detect the effects of JWSNS on stress injury and stress resilience on rats. By TMT cerebrospinal fluid proteomics, bioinformatics analysis and further PRM verification, we aimed to find potential CSF protein targets that play essential roles in JWSNS regulating chronic stress injury and to provide new targets for clinical research.

## Materials and methods

### Animals and treatment groups

**Figure 2.**
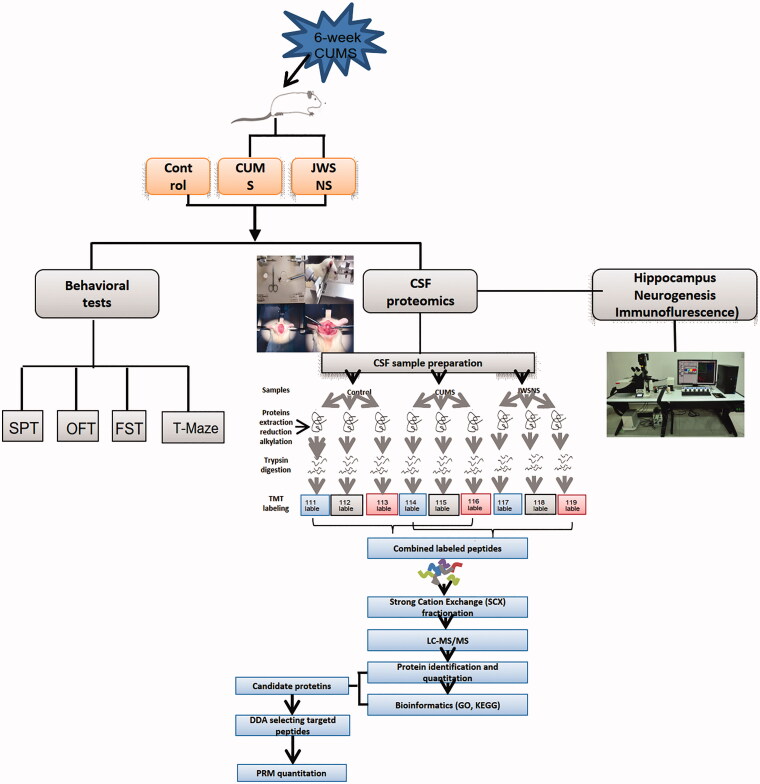
Experiment procedure.

All 54 male Wistar rats weighing 180–220 g (License No. SCXK (Yue) 2018-034) were obtained from the Laboratory Animal Centre of Southern Medical University, Guangzhou, China and housed in cages with the floor covered with sawdust. They were maintained under a 12 h light/dark cycle with the light on between 8:00 and 20:00 at a controlled temperature of 23 ± 2 °C, with food and water available. All efforts were made to minimize animal suffering and all experimental procedures and protocols were approved by the Experimental Animal Ethics Committee of Guangzhou University of Chinese Medicine in China. The Experiment procedure of this study is shown in Figure 2.

After 1-week acclimation, the sucrose preference test was conducted to eliminate unqualified rats. Those with the following performance(s) were excluded: low sucrose preference (<60%), location preference (preferred to drink liquid at a fixed location), drinking too little liquid (neither sucrose solution nor pure water) or excessive drinking (total liquid consumption twice than the average of all rats). All remaining rats were divided into three groups: Control group (CON) (*n* = 14), CUMS group (*n* = 16), and CUMS + JWSNS group (*n* = 17). Rats in CON and CUMS group were given purified water (5 mL/kg, IG), and rats in CUMS + JWSNS group were given JWSNS (16.9 g/kg, IG) twice a day for 6 weeks since the first day of CUMS.

### Drugs

As shown in Table 1, JWSNS decoction was composed of the following dried raw materials with a ratio of 5:15:6:15:5:18:30, root of *Bupleurum chinense* DC. (Chaihu) (Lot No.200201), root of *Paeonia lactiflora* Pall. (Baishao) (Lot No.200401), unripe fruit of *Citrus × aurantium* L. (Zhiqiao) (Lot No.190701), ripe fruit of *Lycium barbarum* L. (Gouqi) (Lot No.191203), ripe fruit of *Gardenia jasminoides* J.Ellis (Zhizi) (Lot No.200401), rootstock of *Rehmannia glutinosa* Seud. (Dihuang) (Lot No.200301) and *Haliotis diversicolor Reeve* (Shijueming) (shell Abalone) (Lot No.190601). Herbs were purchased from Guangzhou ZhixinTang, Guangzhou, China, and authenticated by Professor Jian Wang, School of Basic Medicine, Guangzhou University of Traditional Chinese Medicine. Voucher specimens were deposited at the herbal Herbarium of Chinese Materia Medica College, Guangzhou University of Chinese Medicine, Guangzhou, China. Voucher specimens of the herbal materials were deposited for possible future comparison. The representative fingerprinting chromatograms (Liu et al. 2011) are shown in Figure 1. Above herbs were boiled twice and the decoctions were mixed, filtrated and concentrated to 1.4 kg/L (1 L decoctions equals to 1.4 kg total weight of botanical raw materials) liquidated at 80 °C water bath and stored at 4 °C.

### CUMS procedure

The CUMS protocol was based on our previous studies (Huang et al. 2020). During CUMS modelling, the CON group was housed 4–5 rats per cage with no stressor and the other two groups were solitary-housed and exposed to two arbitrary mild stressors each day. The same stressors were not scheduled for three consecutive days. Stressors included white noise (85 dB, 5 h), thermal swimming (45 °C, 5 min), stroboscopic illumination (300 flashes/min, 5 h), wet bedding (10 h), crowding (four rats within one cage, 10 h), cold swimming (4 °C, 5 min), tail pinching (3 min), restraint (12 h), water deprivation (24 h) and food deprivation (12/24 h). The CUMS procedure lasted for 6 weeks.

### Behaviour Test

#### Sucrose Preference Test (SPT)

The Sucrose Preference Test (SPT) was carried out at the end of the 6-week CUMS exposure. SPT included four phases: sucrose training (48 h), baseline testing (36 h), food and water deprivation (24 h), and sucrose preference testing (12 h). In sucrose training, baseline testing, and sucrose preference testing phase, rats were given the same amount of pure water and 1% sucrose solution. All rats were solitary-housed during SPT. According to the results of Phase 4, sucrose preference (SP) was calculated according to the ratio of sucrose consumption/(water consumption + 1% sucrose consumption) × 100%. Sucrose preference values are used to determine whether a rat was susceptible or resilient.

#### Open Field Test (OFT)

To assess locomotion activity and exploration in a novel environment, Open Field Test (OFT) was performed. Before the test, the rats habituated 1 h in the sound-free and dark behavioural test room. At the beginning of the test, rats were placed individually in the centre of an open black box (100 cm × 100 cm × 48 cm) and then left to explore freely for 5 min. The total distances travelled by rats were recorded by an automatic analysis system (Flydy Co., Ltd, Guangzhou, China). The apparatus was cleaned with detergent prior to each test session to remove any olfactory cues.

#### Forced Swimming Test (FST)

Before the test, experimental rats habituated 1 h in the behavioural test room and were shuffled. A transparent cylindrical pool (30 cm diameter, 100 cm height) was filled with 35 cm deep water (25 ± 1 °C). Rats were forced to swim in the cylindrical pool for 6 min. The duration of immobile posture (rats floating on the surface with limbs immobilized or front paws and tail gently padded to keep head above the water) was considered as the immobility time. The immobility time among the last 4 min was recorded for assessing desperation behaviour in rats. The data were analyzed by a researcher blind to the experimental design.

#### T Maze Test-spatial working memory

The apparatus consists of two closed arms (50 cm × 15 cm) with walls of 15 cm high (goal arms) and one central open arm (70 cm × 15 cm) in dimensions(stem) defining a T shape. The T maze test consists of two phases: orientation phase (day 1), training and testing phase (days 2–5).

On day 1, the rats were trained to be familiar with the apparatus by being placed at the start section and freely exploring in the T-maze for 5 min, with doors open and cheese placed at the end of both goal arms. This step was repeated three times at an interval of 20 min. On days 2–5, the training phase and testing phase were repeated ten times a day at an interval of 20 min. In the training phase, one random goal arm was open and placed with cheese to be found and the other goal arm was closed. In the testing phases, both goal arms were open and only the previous closed arm was placed with cheese. Ten trials were performed for each rat and finding cheese at first try was considered as one right route entry. Accuracy (%) (space perceptive ability) was evaluated as the ratio of the right route (novel route) entries divided by the total number of arm entries, multiplied by 100.

### CSF proteomics

#### CSF extraction

CSF sample was prepared 24 h after the T-maze test. The rats were anaesthetized and exposed to the foramen magnum. intravenous infusion needle (0.45#) was attached to a syringe (1 mL). CSF (about 150 μL per rat) was collected from cisterna magna puncture, centrifuged at 3000 rpm for 15 min at 4 °C. The supernatant of CSF was collected and frozen at −80 °C.

#### Tandem mass tag (TMT) analysis

Protein was extracted from cerebrospinal fluid. After quantification and separation, 30 μL of protein were taken from each sample for proteolysis. TMT labelling was carried out in accordance with the instructions of the TMT labelling kit (Thermo, US). A high pH RP spin column was used for grading. The samples were separated by chromatography (Thermo, USA) and analyzed by mass spectrometry (Thermo, USA). The original data were identified and quantitatively analyzed by Mascot2.2 and Proteome Discoverer1.4. Proteins with a fold change of more than 1.2 or less than 0.83 as well as a statistical *p*-value <0.05 between two groups were selected as DEPs.

#### Bioinformatics analysis

Gene Ontology (GO) mapping and annotation of proteins were conducted using the Blast2GO. Kyoto Encyclopaedia of Genes and Genomes (KEGG) annotation was performed using KAAS (KEGG Automatic Annotation Server). Enrichment analysis was performed by Fisher’s Exact Test, with *p*-values less than 0.05 and FDR value less than 0.5.

#### Parallel reaction monitoring (PRM) quantitative analysis of target proteins

The proteins of CSF samples were extracted and hydrolyzed, and then separated by an HPLC system (Thermo, USA). The separated peptides were analyzed by PRM mass spectrometry (Thermo, USA). The PRM test was repeated three times. Finally, the software Skyline3.7.0 was used to analyze the data of the original PRM file and to quantify the target protein and target peptide.

### Immunofluorescence of the hippocampus DG

#### Tissue preparation

BrdU (Sigma, St. Louis, MO, USA) was intraperitoneally injected 1 week before the sampling date (three injections, 4 h apart, 200 mg/kg), and rats were sacrificed 7 days after the injection. Brains were removed and fixed for 24 h in 4% paraformaldehyde at 4 °C and then dehydrated in 30% sucrose at 4 °C until sinking at the bottom. According to the coronary sulcus, serial coronal sections (40 µm) containing the dentate gyrus of hippocampus from Bregma 2.7 mm to 6.7 mm were cut using a freezing microtome (Leica SM2000R, Richmond Hill, Ontario, Canada) and stored at −20 °C.

#### BrdU/DCX double labeling

To examine the cell proliferation and differentiation, double immunofluorescence staining for BrdU and DCX was performed. Sections were treated with 2 NHCL for 20 min at 37 °C, rinsed in borate buffer (0.1 M, pH 8.4), blocked in 5% goat serum (containing 0.03% Triton-X-100) at room temperature for 1 h and incubated with rat anti-BrdU antibody (1:150, mAb, Abcam) and rabbit anti-DCX antibodies (1:150, Abcam) overnight at 4 °C. After rinsing in Tris-buffered saline (TBS) with 0.01% Tween-20, the sections were incubated with AlexaFluor^®^488 goat anti-rabbit (1:500, Abcam) and AlexaFluor^®^594 goat anti-rat antibodies (1:500, Abcam) at 37 °C for 2 h. After rinsing with Tris-buffered saline with 0.01% Tween-20 and DAPI staining, images were captured using a laser scanning confocal microscope (LSM800, ZEISS, Oberkochen, Germany).

#### NeuN labeling

NeuN was used to label mature neurons and to reflect the number of neurons in DG. The sections were rewarmed, membrane-ruptured with Triton-X, blocked with goat serum, and incubated with rabbit anti-NeuN antibody (1:1000, Abcam, UK) overnight at 4 °C. After being washed in TBST, the sections were incubated with AlexaFluor^®^ 488 goat anti-rabbit (1:500, Abcam, UK) at 37 °C for 2 h. After being washed in TBST and DAPI staining, images were captured by a laser confocal microscope (LSM800, ZEISS, Germany). The ratio of NeuN positive cells (%) = NeuN positive cell number/total number of nuclei × 100%.

### Data analysis

The data were statistically analyzed using SPSS 22.0 Software (IBM, Armonk, IL, USA). The data of each group were consistent with a normal distribution (Shapiro-Wilk test). An independent samples *t*-test was used for comparisons between two groups with homogeneity of variance, and one-way analysis of variance was used for comparisons of three or more groups. In the pairwise comparisons, the least significant difference test was used to assess results exhibiting homogeneity of variance, and the Games-Howell method was used to assess results with the heterogeneity of variance. All results are expressed as the mean ± SEM. *p*-Values <0.05 were considered statistically significant.

## Results

### Behaviour tests

#### Anhedonia in SPT

**Figure 3.**
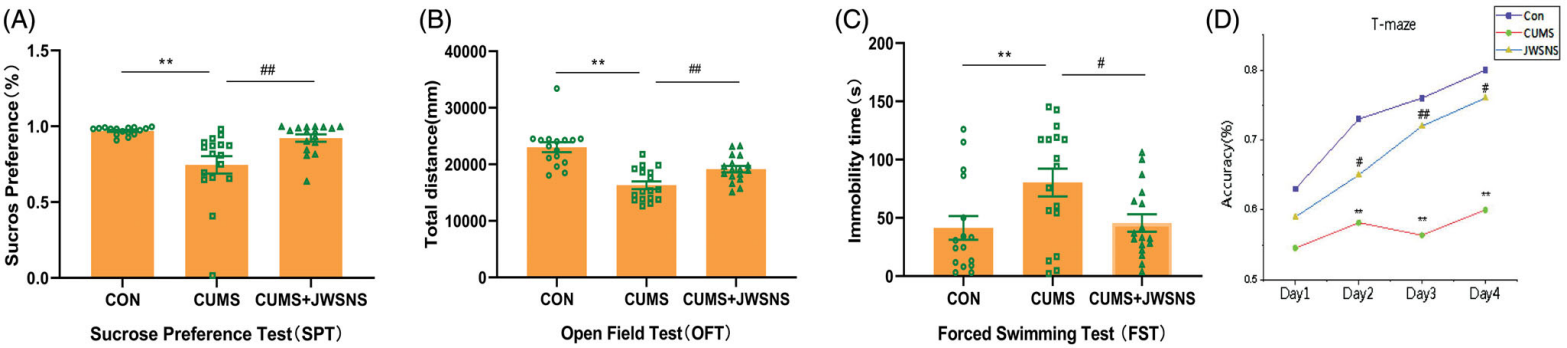
Behavioural test results. (A) Result of Sucrose Preference Test (SPT); (B) result of Open Field Test (OFT); (C) result of Forced Swimming Test (FST); (D) result of T-Maze. Data were expressed as the mean ± SEM, *p < 0.05, **p < 0.01, #*p* < 0.05, ##*p* < 0.01.

Sucrose preference test (SPT) was conducted to stimulate anhedonia. Rats with mental stress damage tend to perform poorly in SPT. Before CUMS, SPT of rats in different groups showed no significant difference. As showed in Figure 3(A), rats in CUMS (74.59 ± 5.73%) acted worse than those in CON group (96.99 ± 0.65%, *p <* 0.01) while rats in CUMS + JWSNS group (92.24 ± 2.39%) showed more significant sucrose preferences than those in CUMS group (*p* < 0.01).

**Figure 4.**
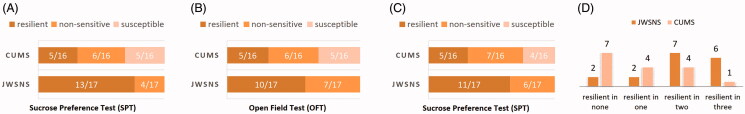
Amount of resilient, susceptible, non-sensitive rats of CUMS and JESNS group in Sucrose Preference Test (SPT) (A), Open Field Test (OFT) (B) and Forced Swimming Test (FST) (C); and (D) amount of rats of CUMS and JWSNS group that showed resilience in one, two and all above behavioural tests.

According to results of the CUMS group, SP was ranked from highest to lowest, with the top 1/3 being resilient rats (SP > 87.2%, *n* = 5) and the bottom 1/3 being susceptible rats (SP < 70%, *n* = 5), with 13/17 of resilient rats, 0/17 of susceptible rats, and 4/17 of insensitive rats after JWSNS intervention (Figure 4(A)).

#### Locomotion activity in OFT

To explore the experimental animals’ spontaneous exploratory behaviour, the total distance travelled by rats in the OFT was measured. Figure 3(B) shows that CUMS markedly reduced the total distance (23,033 ± 891.5 mm vs 16,303 ± 689.3 mm, *p* < 0.01) as compared to the CON group. Treatment of JWSNS (19,147 ± 569.2 mm, *p* < 0.01) significantly reversed the reduction as compared to CUMS group. A third of rats in CUMS group with the longest total distance (> 18616.12 mm) were considered stress-resilient and a third of rats in CUMS group with the shortest total distance (<14,044 mm) were stress susceptible. While in CUMS + JWSNS group, ten of seventeen rats were stress-resilient, seven were non-sensitive and none was susceptible. It indicated that JWSNS could increase the ratio of stress-resilient rats (Figure 4(B)).

#### Desperation in FST

Freezing time in the forced swimming test (FST) indicates the degree of the desperation of rats. The longer the rats froze in FST, the more desperate the rats were. Figure 3(C) shows that the freezing time of CUMS group (80.35 ± 11.94 s) was significantly longer as compared to CON (41.46 ± 10.16 s, *p* < 0.01) and CUMS + JWSNS group (45.57 ± 7.49 s, *p* < 0.05).

Rats with the longest immobility time (>118 s) (*n* = 4) in CUMS group were considered as susceptible and the shortest (<56 s) (*n* = 5) were considered as stress-resilient. After preventive treatment of JWSNS, 11 of 17 rats were stress-resilient and 6 of 17 were non-sensitive to stress (none was susceptible) (Figure 4(C)). The amount of stress-resilient rats was increased by JWSNS.

Rats that showed stress resilience and susceptibility in the above behavioural tests were analyzed (Figure 4). As shown in Figure 4(D), one rat in CUMS group was stress-resilient in three behavioural tests, four in two, four in one and seven were stress-resilient in none; as in CUMS + JWSNS group, 6 were stress-resilient in three tests, 7 in 2 and 2 in 1.

#### Spatial cognitive ability in T-maze

By using repeated measure ANOVA (CUMS group by the accuracy of T-maze at four-time points), we found no interaction effects. Accuracy in T-maze was associated with the spatial cognitive ability of rats. As showed in Figure 3(D), accuracy (days 2–4) of rats in CUMS group was significantly lower than CON group (*p* < 0.05), and the accuracy (days 2–4) of rats in CUMS + JWSNS group was higher than CON group (*p* < 0.05).

### Proteomics screening results in CSF

To identify proteins (in CSF) related to CUMS stimulus and JWSNS, which may play a critical role in the pathological and improvement process of mental stress damage, the quantitative proteomics technique Tandem Mass Tag (TMT) technology was adopted. As result, 1935 proteins were screened/identified in rat CSF. Significantly differentially expressed proteins (DEP) were selected according to the standard of the ratio (CON/CUMS or CUMS/JWSNS) > 1.2 and *p*-value <0.05.

### Differential expression of proteins in CSF screened by TMT before and after the CUMS

**Figure 5.**
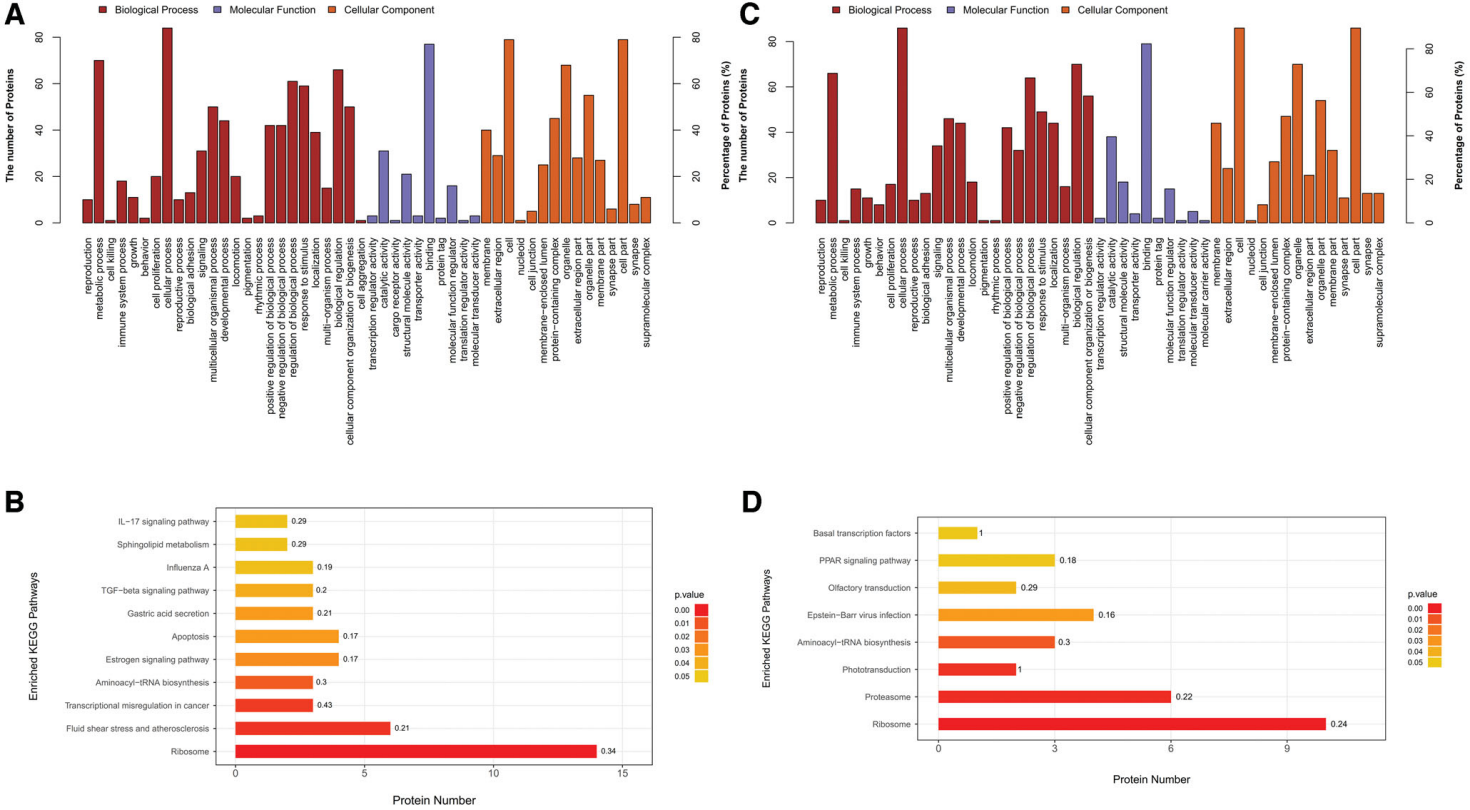
GO annotation and KEGG pathway enrichment analysis of Differently expressed proteins (DEPs). (A) GO annotation of DEPs in CON and CUMS group; (B) KEGG pathway enrichment analysis of DEPs in CON and CUMS group; (C) GO annotation of DEPs in CUMS and JWSNS group; (D) KEGG pathway enrichment analysis of DEPs in CUMS and JWSNS group.

Results show that a total of 100 DEPs were detected between samples of CON and CUMS group, in which 34 were up-regulated and 66 down-regulated. To better understand the biological function of DEP, an enrichment analysis of the GO and Kyoto Encyclopaedia of Genes and Genomes (KEGG) pathway was performed. The results of the GO annotation of these 100 proteins (Figure 5(A)) show that these proteins are involved in binding, catalytic activity, structural molecule activity, molecular function regulator and transcription regulator activity. In terms of molecular function, they mainly possess binding, catalytic and transporter activity. The result of the KEGG pathway annotation indicates that these 100 DEPs are mostly involved in cellular processes, metabolic processes, biological regulation, regulation of biological process and response to the stimulus.

### Differentially expressed proteins in CSF regulated by JWSNS

Results show that a total of 96 DEPs were detected between samples of CUMS and CUMS + JWSNS group, in which 33 were up-regulated and 63 down-regulated. GO annotation (Figure 5(C)) shows that these proteins are involved in binding, catalytic activity, structural molecule activity, molecular function regulator and molecular transducer activity. The result of the KEGG pathway annotation (Figure 5(D)) indicates that these 96 differentially expressed proteins are mostly involved in cellular process, biological regulation, metabolic process, regulation of biological process and cellular component organization or biogenesis.

### Significantly differentially expressed proteins regulated by both CUMS and JWSNS

**Figure 6.**
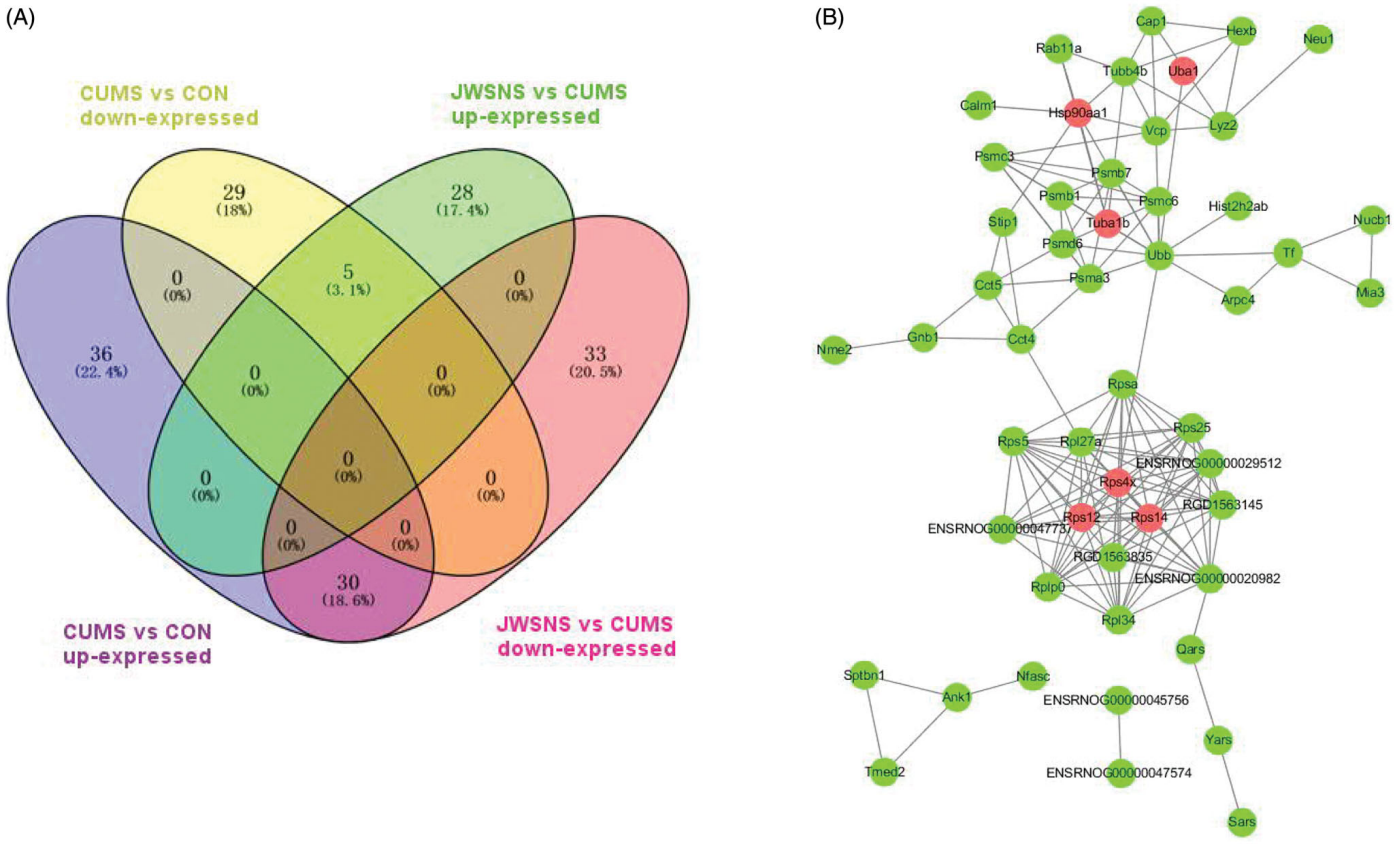
(A) Venn map of differentially expressed proteins co-regulated by JWSNS and CUMS; (B) Protein–Protein Interaction Network (PPI) of co-regulated DEPs.

Thirty-five DEPs were co-regulated by CUMS and JWSNS, in which 30 up-regulated by CUMS, down-regulated by JWSNS and 5 down-regulated by CUMS, up-regulated by JWSNS (Table 2, Figure 6(A)). KEGG pathway annotation indicates that these 35 differentially expressed proteins are mostly involved in the ribosome, Aminoacyl-tRNA biosynthesis, Epstein-Barr virus infection, fluid shear stress and atherosclerosis, oestrogen signalling pathway, pathways in cancer, PI3K-Akt signalling pathway, proteasome, and tuberculosis.

**Table 2.** Differentially expressed proteins co-regulated by JWSNS and CUMS.

Protein IDs	protein_name	gene_id	gene_name	CUMS vs JWSNS	CUMS vs CON
ENSRNOP00000069539	MIA SH3 domain ER export factor 3-	ENSRNOG00000059202	AABR07021988.1	1.667021	1.406647
ENSRNOP00000014807	Insulin-like growth factor binding protein 6	ENSRNOG00000010977	Igfbp6	1.240446	1.284354
ENSRNOP00000012293	Alpha-2u globulin PGCL3	ENSRNOG00000009273	LOC259244	1.289872	1.277781
ENSRNOP00000027860	HtrA serine peptidase 1	ENSRNOG00000020533	Htra1	1.227982	1.246935
ENSRNOP00000004764	EGF containing fibulin extracellular matrix protein 1	ENSRNOG00000003553	Efemp1	1.239637	1.212169
ENSRNOP00000033950	Ubiquitin-like modifier activating enzyme 1	ENSRNOG00000019164	Uba1	0.607629	0.787971
ENSRNOP00000017234	Heparin binding growth factor	ENSRNOG00000042261	Hdgf	0.526065	0.771831
ENSRNOP00000004867	Small ubiquitin-like modifier 2	ENSRNOG00000003670	Sumo2	0.558033	0.746357
ENSRNOP00000074688	Ubiquitin C	ENSRNOG00000057823	Ubc	0.667817	0.733036
ENSRNOP00000015598	RAB11a, member RAS oncogene family	ENSRNOG00000011302	Rab11a	0.70795	0.730942
ENSRNOP00000009556	heat shock protein HSP 90-alpha	ENSRNOG00000007219	LOC103692716	0.675043	0.730779
ENSRNOP00000024430	Vimentin	ENSRNOG00000018087	Vim	0.797659	0.727629
ENSRNOP00000009649	Proteasome 26S subunit, ATPase 6	ENSRNOG00000007203	Psmc6	0.620382	0.718329
ENSRNOP00000001518	Ribosomal protein lateral stalk subunit P0	ENSRNOG00000001148	Rplp0	0.656478	0.696513
ENSRNOP00000072016	TATA-box binding protein associated factor 15	ENSRNOG00000058393	Taf15	0.667331	0.693424
ENSRNOP00000070868	Tubulin, alpha 1B	ENSRNOG00000053468	Tuba1b	0.588112	0.689164
ENSRNOP00000026696	Heat shock protein family A member 9	ENSRNOG00000019525	Hspa9	0.535606	0.679796
ENSRNOP00000019247	Ribosomal protein L27a	ENSRNOG00000014214	Rpl27a	0.692805	0.673251
ENSRNOP00000071233	Spectrin, beta, non-erythrocytic 1	ENSRNOG00000005434	Sptbn1	0.736569	0.670589
ENSRNOP00000022603	Calmodulin 3	ENSRNOG00000016770	Calm3	0.526308	0.636832
ENSRNOP00000010674	Tyrosyl-tRNA synthetase	ENSRNOG00000007213	Yars	0.54097	0.556245
ENSRNOP00000009249	Proteasome 26S subunit, non-ATPase 6	ENSRNOG00000006751	Psmd6	0.46405	0.550629
ENSRNOP00000056260	40S ribosomal protein S14-like	ENSRNOG00000018774	LOC100911847	0.534893	0.523183
ENSRNOP00000038448	Seryl-tRNA synthetase	ENSRNOG00000020255	Sars	0.479899	0.517425
ENSRNOP00000071398	Glutaminyl-tRNA synthetase	ENSRNOG00000060912	Qars	0.587713	0.514999
ENSRNOP00000070867	Neuraminidase 1	ENSRNOG00000032942	Neu1	0.550484	0.48937
ENSRNOP00000001397	Transmembrane p24 trafficking protein 2	ENSRNOG00000001053	Tmed2	0.446784	0.473675
ENSRNOP00000070331	SET nuclear proto-oncogene	ENSRNOG00000025892	Set	0.431938	0.455886
ENSRNOP00000026528	Ribosomal protein S5	ENSRNOG00000019453	Rps5	0.354136	0.414189
ENSRNOP00000034657	Similar to Finkel-Biskis-Reilly murine sarcoma virusubiquitously expressed	ENSRNOG00000047365	LOC687780	0.401339	0.392441
ENSRNOP00000033144	Ribosomal protein s25	ENSRNOG00000027503	Rps25	0.320287	0.364721
ENSRNOP00000004278	Ribosomal protein S4, X-linked	ENSRNOG00000003201	Rps4x	0.3718	0.353676
ENSRNOP00000022184	Ribosomal protein S12	ENSRNOG00000016411	Rps12	0.227142	0.255554
ENSRNOP00000060949	Ribosomal protein L34	ENSRNOG00000016387	Rpl34	0.145147	0.150869
ENSRNOP00000074005	Dyskerin pseudouridine synthase 1	ENSRNOG00000055562	Dkc1	0.153169	0.13148

### PRM verification

Based on the results of GO annotation and KEGG enrichment analysis, we focussed on six significantly different proteins at key nodes in the protein-protein interaction network (Figure 6(B)), namely Tuba1b, Rps14, Rps4x, Rps12, HSP90AA1, and Uba1. They are involved in apoptosis, neuron proliferation, differentiation, growth and development. PRM results (Table 3) showed that they were up-regulated by CUMS and down-regulated by JWSNS, which were consistent with TMT results.

**Table 3.** PRM quantitative analysis of target proteins.

Protein name	Gene name	PRM results		TMT results	
		CUMS/CON	JWSNS/CUMS	CUMS/CON	JWSNS/CUMS
ribosomal protein S4, X-linked	Rps4x	3.4306	0.6859	2.8274	0.3718
heat shock protein HSP 90-alpha	HSP90AA1	3.6841	0.2989	1.3684	0.6750
ribosomal protein S12	Rps12	6.4356	0.3992	3.9131	0.2271
ubiquitin-like modifier activating enzyme 1	Uba1	2.3242	0.5349	1.2691	0.6076
ribosomal protein S14	Rps14	15.7618	0.1425	1.9114	0.5349
tubulin, alpha 1B	Tuba1b	2.8018	0.6105	1.4510	0.5881

### Hippocampal dysfunction alleviated by JWSNS

#### JWSNS reverted the neurogenetic disorder in DG induced by CUMS

**Figure 7.**
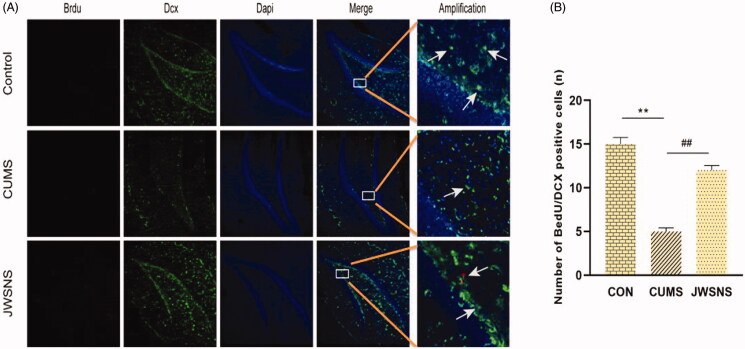
(A) BrdU/DCX positive cells in DG, red: BrdU, green: DCX, blue: DAPI. (B) Number of BrdU/DCX positive cells in DG (*n* = 5 per group). Data were expressed as the mean ± SEM. *p < 0.05, **p < 0.01, vs. control. #*p* < 0.05, ##*p* < 0.01, vs. CUMS.

Adult neurogenesis is a common feature of the dentate gyrus in mammals and is divided into three main parts: cell proliferation, neuronal differentiation and cell survival. In the immunofluorescence experiment, Brdu/Doublecortin (DCX) co-labelling new nerve cells and neurons were used to reflect the number of neural precursor cells in the DG area of the hippocampus. The decrease of positive cells indicated that the proliferation and differentiation ability of neural stem cells reduced and neurogenesis dysfunction. As shown in Figure 7, compared with the CON group, the number of BrdU/DCX positive cells in DG of the CUMS group reduced significantly (*p* < 0.05). Compared with the CUMS group, JSWNS significantly increased the number of BrdU/DCX positive cells in DG (*p* < 0.01). It indicates that JWSNS treatment could alleviate CUMS-induced neurogenesis dysfunction in DG.

#### Effect of JWSNS on relatively number of neurons in DG in CUMS rats

**Figure 8.**
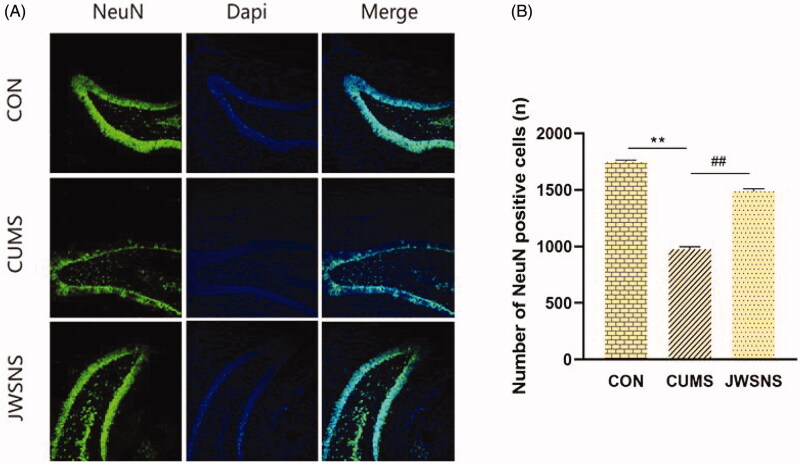
(A) NeuN positive cells in DG, green: NeuN, blue: DAPI. (B) Number of neurons in DG (*n* = 5 per group). Data were expressed as the mean ± SEM. *p < 0.05, **p < 0.01, vs. control. #*p* < 0.05, ##*p* < 0.01, vs. CUMS.

As shown in Figure 8, compared with the CON group, the relative number of neurons in the DG of the CUMS group were reduced significantly (*p* < 0.01). Compared with the CUMS group, JWSNS significantly increased the relative number of neurons (*p* < 0.01) suggesting that JWSNS treatment could resist a neuronal reduction in DG.

## Discussion

To explore anti-stress injury mechanism of JWSNS, a 6-week CUMS model and simultaneous JWSNS intervention were applied in this study. In behaviour tests, CUMS-rats showed low locomotor activity, anhedonia, desperation and decreased learning and memory ability and JWSNS had a positive effect on them by increasing the percentage of resiliency rats and reducing susceptible rats. It indicated that JWSNS could prevent stress-related psychiatric disorders by alleviating stress-induced symptoms, improving stress resilience and reducing stress susceptibility.

Up till now, evaluation criteria of stress resilience and susceptibility (no matter in human or animal studies) considered only behavioural changes obtained from the questionnaire or behavioural tests. Numerous studies have shown that about one-third of MDD or PTSD patients have less severe clinical symptoms and show stress resilience (Russo et al. 2012). In animal experiments, the researchers found that about a third of mice did not exhibit behavioural manifestations such as social avoidance after social failure. They showed only a certain degree of social inability and significant adaptability to other chronic social stresses (Russo et al. 2012). In this study, 1/3 of rats in CUMS that performed best and rats in the CUMS + JWSNS group with similar performance were defined as resilient rats. This criterion was applied in OFT, SPT and FST. Results of T-maze were not included because continuous data (accuracy) were impossible to be differentiated by this criterion. In many reports, a single behavioural test (usually SPT or social defeat test) was used as evaluation criteria of stress resilience and susceptibility. In order to assess stress resilience and susceptibility in different behavioural phenotypes on multiple levels, resilience and susceptibility of each rat in three different behavioural tests were considered as evaluation criteria in this study. Results showed that after JWSNS intervention more rats were resilient in two or three behavioural tests (Figure 4(D)). It was suggested that JWSNS could increase stress resilience in different behavioural phenotypes and partly demonstrate that JWSNS could affect multiple targets of the stress response. The results provided more systematic and accurate evidence for JWSNS regulating stress resilience. Besides locomotion activity, despair and learning and memory ability we observe in this study, anxiety, social dysfunction and fear are as well important and regular mental stress response and ought to be included in a multi-dimensional evaluation system of stress resilience and susceptibility, which is required no matter in preclinical or clinical experiments.

Unlike normal was administration of antidepressants and anti-anxiety drugs, JWSNS was administered during rather than after CUMS modelling, meaning JWSNS was intended not only to treat stress damage but also to intervene in the intermediate process of the stress response. Nowadays, we have limited options to deal with mental illness. Prevention measures include mental health education, like mental health courses in school, psychological counselling and higher attention paid to the psychological state of workers by enterprises. Therapeutic options range from drugs to psychological counselling after diagnosis. It is undeniable that early mental health education and psychological counselling could only benefit a limited number of people because of high fees and cultural influence. Especially in Asia, people are often reluctant to face and deal with psychological and mental problems when they were raised to be tough and repressed. As for drugs, compliance, treatment-resistant, withdrawal symptoms and side effects of antidepressant and antianxiety drugs remain unsolved (Haddad 2001). Compared with the therapeutic effect of drugs such as parosidine, the Chinese medicine compound such as JWSNS emphasis more on adjusting stress resilience and preventing stress injury. The dosage regimen similar to health care products is also in line with the need for disease prevention. Numerous studies have shown that complementary and alternative therapies (CAM) are widely used in mental disorders such as depression and anxiety disorders. Herbal remedies are most commonly used (Liu et al. 2015). As a classic Chinese medicine agent, JWSNS has a long history and has been widely used in the clinical intervention in mental illness for thousands of years, and its anti-stress injury effect has been repeatedly confirmed in preclinical and clinical studies. We believe that JWSNS could play an important role in the prevention of mental illness after a major disaster and in the daily mental health care of stressed populations (stressed people) and that the use of these scenarios can also increase the clinical credibility of the compound and significantly reduce social medical costs.

Considering the wide range of stress damage and Chinese medicine compound sites, cerebrospinal fluid is an ideal research object to explore the possible proteins and pathways of JWSNS regulating stress. Through high-throughput proteomics and PRM quantitative analysis, six different expression proteins (DEPs) that played an important role in the JWSNS anti-stress injury process were selected – Tuba1b, Rps14, Rps4x, Rps12, HSP90AA1, Uba1 was up-regulated by CUMS and down-regulated by JWSNS intervention. These six DEPs are mainly related to apoptosis, proliferation and cell stability in biological function.

Ribosomal proteins are closely related to complex functions – coordinating protein biosynthesis to maintain homeostasis and survival in cells. Many ribosomal proteins have secondary functions independent of their involvement in protein biosynthesis. Many of these proteins act as cell proliferation regulators and, in some cases, as inducers of cell death. Some scholars (Hetman and Slomnicki 2019) have suggested that ribosome biogenesis disorder may lead to insufficient proliferation and/or loss of neural precursor cells and apoptosis of immature neurons. In this study, a total of 35 differential proteins co-regulated by CUMS and JWSNS were screened, among which 8 were ribosome proteins, represented by Rps14, Rps4x and Rps12. They were highly co-expressed in the CUMS group, and their expression was reduced by JWSNS. These ribosomal proteins are highly differentiated and are key nodes in the protein interaction network, indicating that they are important proteins involved in the stress damage regulation of JWSNS. Consistent with our results, Hiroaki et al. (Hori et al. 2018) found that the ribosome gene was up-regulated in depression, bipolar disorder and schizophrenia and was associated with stress susceptibility in the analysis of gene expression profiles in human peripheral blood.

Highly conserved tubulin-β and tubulin-α assemble into dynamic microtubules that perform a variety of important cellular functions such as structural support, transport and stress generation in cell division. As cellular partners of HtrA3, tubulin-β, actin and TCP1 protein may influence cytoskeletal dynamics. Tubulin plays an important role in the nervous system. Mutations in tubulin-αβ dimers and tubulin-γ can cause brain malformations and impair cognitive function (Keays et al. 2007). In this study, levels of tubulin-β in cerebrospinal fluid of CUMS rats were increased, while those in the CUMS + JWSNS group were down-regulated. It was suggested that the increase of tubulin-β in CSF of CUMS rats may be related to the death of neurons and gliocyte, while the compensatory increase of cytoskeleton may be related, which was not observed in CUMS + JWSNS group.

Heatshock protein 90α(Hsp90α), encoded by the HSP90AA1 gene, is the stress-inducible isoform of the molecular chaperone Hsp90. Hsp90α is regulated differently and has different functions when compared to the constitutively expressed Hsp90β isoform, despite high amino acid sequence identity between the two proteins. The processes associated with Hsp90α are more related to adaptation to stress or other specialized functions. In the presence of cellular stress, Hsp90α levels increase (Zuehlke et al. 2015). Proteomics results of TMT and PRM both show that Hsp90α is up-regulated in CUMS group and down-regulated in CUMS + JWSNS group. Hsp90 is widely expressed in all regions of the adult mammalian nervous system and preferentially localized on neurons (D’Souza and Brown 1998). Most studies have shown that Hsp90 is involved in neurodegenerative diseases, neuron differentiation and axon outgrowth (Benitez et al. 2014). Thus, gives us sufficient evidence to make the inference that JWSNS could facilitate neurogenesis through regulating Hsp90α expression.

UBA1 is an essential and highly conserved protein in eukaryotes, encoded by the UBE1 gene (Xp11.3) on the Human X chromosome (Handley-Gearhart et al. 1994; Lv et al. 2018). Our proteomics results showed that Uba1 expression was decreased under CUMS and JWSNS restored the activity of Uba1 in the cerebrospinal fluid of stress-injured rats. Uba1 is a major E1 ubiquitin activase in mammalian cells and plays a role at the top of ubiquitin activation and conjugated cascade. It controls not only protein hydrolysis but also the cell cycle process, DNA damage repair, transcription, translation, vesicle transport and apoptosis. In the nervous system, UBA1 activity and ubiquitination are more broadly involved in the regulation of many aspects of neural function, such as neuronal differentiation, growth and development, neuronal excitability, neurotransmission, long-term enhancement (LTP), and synaptic formation and elimination (Mabb and Ehlers 2010; Kawabe and Brose 2011). Uba1 damage could lead to neuronal dysfunction and degeneration.

It is clear the six DEPs mainly involve cell proliferation, differentiation and survival. Interestingly, neuron and gliocyte apoptosis, neural stem cell proliferation and differentiation and gliocyte proliferation are the common basic pathological manifestations of psychological stress injury. Therefore, we presume that JWSNS may play an anti-stress injury role by regulating the six DEPs. For further verification, the hippocampus DG region was chosen to observe the effects of JWSNS based on the following considerations:

First, the hippocampus is essential in regulating learning, memory, emotional behaviour and is sensitive to stress. Brain imaging studies showed that the volume of the hippocampus was associated with the severity of stress damage. Similar results were observed in animal experiments – hippocampus shrank and hippocampal-dependent learning and memory ability declined (Schoenfeld and Gould 2012). Second, neurogenesis includes the proliferation, migration, differentiation and survival of neural precursor cells. Adult neurogenesis occurs only in subventricular zone (SVZ) and subgranularzone (SGZ) of the hippocampus dentate gyrus area. Stress has been shown to have a predominantly negative impact on adult neurogenesis in the dentate gyrus of the hippocampus. In particular, chronic exposure to stressful situations including psychosocial or physical stressors has been demonstrated to be detrimental to adult neurogenesis in the DG in mammals (Heine et al. 2004; Boku et al. 2018). Last, cerebrospinal fluid contacts with neural precursor cells on the surface of the central system directly and provides neurogenic signals that promote growth and survival for neural precursor cells in SGZ – inducing neural precursor cell proliferation and neuron differentiation). Studies have found that reduced neurogenic effects of cerebrospinal fluid (changes in cerebrospinal protein and reduced neurogenic induction) could lead to a decrease in neurogenic activity in the adult DG region (Gato et al. 2020).

The immunofluorescence results indicated neurogenesis dysfunction and neuron loss in DG region in CUMS group, which were basic pathological changes of brain cell proliferation, differentiation and survival disorders of stress-damaged brain cells and repair effect of JWSNS, which was consistent with the results of the T maze – indicating that JWSNS could improve hippocampus-related learning and memory ability damaged by stress.

This study shows the pharmacological effects of JWSNS in the hippocampus DG region – regulating the proliferation, differentiation and survival of neurons during psychological stress. But whether the six DEPs (Tuba1b, Rps14, Rps4x, Rps12, HSP90AA1, Uba1) play a direct role in hippocampus requires further verification. For instance, detecting the expression and function of the above proteins in the hippocampus in future studies might be helpful.

## Conclusions

Although the specific mechanism still needs further comprehensive study, available evidence showed that JWSNS could effectively intervene in neuropsychological diseases with pathological manifestations mainly of hippocampus neuron damage. It is certain that researchers would pay more attention to prevention rather than treatment of stress-related diseases. In addition to the role of social psychology in early mental health education, Chinese medicine could also play an important role in improving stress resilience and preventing mental illness. In addition, in the exploration of the mechanism of Chinese medicine on the brain such as the hippocampus, CSF and peripheral system could be considered as the intermediate way.

## Author contributions

Study conception: Li-Li Wu (wulili@gzucm.edu.cn); Study design: Li-Li Wu (wulili@gzucm.edu.cn) and Can Yan (yc1970@gzucm.edu.cn); Manuscript writing: Han-Zhang Wang (20193101004@stu.gzucm.edu.cn) and Wu-Long Luo (20181104420@stu.gzucm.edu.cn); Manuscript revision: Ning-Xi Zeng (20182104110@stu.gzucm.edu.cn), Ling-Li (20181104427@stu.gzucm.edu.cn); Experiment and data analysis: Han-Zhang Wang (20193101004@stu.gzucm.edu.cn), Wu-Long Luo (20181104420@stu.gzucm.edu.cn), Ning-Xi Zeng (20182104110@stu.gzucm.edu.cn), Hui-Zhen Li (20193101001@stu.gzucm.edu.cn) and Li-Li Wu (wulili@gzucm.edu.cn). All authors approved the final version of the paper.

## Data Availability

The mass spectrometry proteomics data have been deposited to the ProteomeXchange Consortium via the PRIDE partner repository with the dataset identifier PXD021735. The mass spectrometry proteomics data have been also deposited to the iProX with the dataset identifier PXD021776.
